# The capacity limitations of multiple‐template visual search during task preparation and target selection

**DOI:** 10.1111/psyp.14720

**Published:** 2024-11-03

**Authors:** Anna Grubert, Ziyi Wang, Ella Williams, Mikel Jimenez, Roger Remington, Martin Eimer

**Affiliations:** ^1^ Department of Psychology Durham University Durham UK; ^2^ Department of Psychiatry Oxford University, Warneford Hospital Oxford UK; ^3^ Department of Psychology University of Minnesota Minneapolis Minnesota USA; ^4^ Department of Psychological Sciences, Birkbeck University of London London UK

**Keywords:** attentional control, attentional templates, capacity limitations, event‐related potentials, N2pc, visual search

## Abstract

Visual search is guided by mental representations of target‐defining features (attentional templates) that are activated in a preparatory fashion. It remains unknown how many templates can be maintained concurrently, and what kind of costs are associated with multiple‐template versus single‐template search. Here, we compared the operation of attentional templates during three‐color and single‐color search tasks. Preparatory template activation processes were tracked by measuring N2pc components to task‐irrelevant singleton color probes that appeared in rapid succession during the interval between search displays. These probes attract attention (as indexed by an N2pc) if the corresponding color template is active at the time when the probe appears. In a three‐color search task where target identity was fully predictable (Experiment 1), only probes that matched the upcoming target color triggered N2pcs, demonstrating that only a single target template was activated. When three possible color targets appeared randomly and unpredictably (Experiment 2), probes that matched any of these colors triggered N2pcs, demonstrating that all three templates were activated concurrently. However, relative to a single‐color search task, clear costs emerged in this three‐color task for attentional guidance toward search targets and for search performance. These costs appear to be linked to inhibitory interactions between simultaneously active search templates. These findings show that while at least three target templates can be maintained in parallel, multiple‐template search is still subject to capacity limitations which affect both template‐guided attentional guidance and the subsequent selective processing of search targets.

## Abstract 2

The mechanisms of search preparation during high‐load search at working memory capacity are not very well understood. We used electrophysiological markers of attentional selection to show that participants can prepare for three colors simultaneously if they must, for example, because target colors appear randomly. However, if target colors alternate predictably, participants make use of strategic opportunities to reduce working memory load and only activate the one color template that is directly relevant for the upcoming search episode.

## 1 INTRODUCTION

In visual search, knowledge about the properties of target features enables observers to guide their attention in a goal‐directed fashion. Representations of target properties (attentional templates) bias visual processing in favor of objects with template‐matching features, so that these objects are more likely to be detected, attended, and identified than non‐matching distractors. Because target templates are assumed to be held in visual working memory (Carlisle et al., 2011; Eimer, 2014; Olivers et al., 2011), it should in principle be possible to activate multiple‐target templates simultaneously, until working memory capacity (typically 3–4 items; e.g., Cowan, 2001) is exceeded. Employing multiple templates in parallel would be particularly useful in tasks where observers search for one of several possible target objects.

The capacity of template‐guided visual search, and the nature of any capacity limitations in this domain, have recently become the object of intense study (see Ort & Olivers, 2020, for a review). While it has been argued that only a single attentional template can be maintained at any given time (e.g., Houtkamp & Roelfsema, 2009; Olivers et al., 2011), there is now strong behavioral and electrophysiological evidence for multiple‐target template activation (e.g., Beck et al., 2012; Berggren & Eimer, 2019; Grubert et al., 2016; Grubert & Eimer, 2016, 2023; Irons et al., 2012; Kerzel & Grubert, 2022; Moore & Weissman, 2010; Ort et al., 2019). While it seems clear that at least two target templates can be maintained simultaneously, multiple‐target search is typically less efficient than searching for a single constant target object (see Ort & Olivers, 2020, for a summary). These costs indicate that some capacity limitations arise when several attentional templates are activated at the same time.

Because attentional templates for known target features are activated in a preparatory fashion, insights into possible capacity limitations of template activation can be obtained by investigating these processes prior to the start of a particular search episode. We have recently developed a new method that employs EEG markers to track the activation states of target templates in real‐time during search preparation (Grubert & Eimer, 2018). In our rapid serial probe presentation (RSPP) paradigm, participants search for color‐defined target objects among multiple distractors and irrelevant probe displays are presented rapidly (every 200 ms) during the interval between successive search displays. Each of these probe displays includes a color singleton that either matches the color of the target or a distractor color. Any attentional capture by target‐matching probes indicates that a corresponding color template is active at the moment when the probe is presented. To measure such probe‐induced attentional capture, we recorded EEG during task performance and computed N2pc components for each individual probe position between two search displays. The N2pc is a negative event‐related potential (ERP) component, triggered at posterior scalp electrodes contralateral to objects with task‐relevant features around 180–200 ms after stimulus onset, that reflects the rapid allocation of attention to candidate target objects (e.g., Eimer, 1996; Luck & Hillyard, 1994; Woodman & Luck, 1999). The rationale for such probe‐related attentional capture builds on the task‐set contingent capture literature, in which N2pc components were measured in response to template matching, but task‐ irrelevant, cues that preceded search onset (e.g., Barras & Kerzel, 2016; Eimer & Kiss, 2008; Goller et al., 2020; Grubert & Eimer, 2016; Livingstone et al., 2017; Sawaki & Luck, 2013; Schönhammer et al., 2020).

During the search for a constant color‐defined target (Grubert & Eimer, 2018), singleton probes that matched the current target color triggered N2pc components from about 1000 ms prior to the onset of the next search display, indicating that a color‐selective target template was activated in a transient fashion during the preparation for search. In contrast, no such N2pcs were elicited by singleton probes in a distractor color, demonstrating that N2pcs to target‐color probes did not merely reflect salience‐driven attentional capture. To investigate whether multiple preparatory target templates can be activated concurrently, a follow‐up study employed the same RSPP procedures, except that two possible color‐defined search targets now alternated between trials in a fully predictable fashion between successive search displays (ABAB; Grubert & Eimer, 2020). Reliable N2pcs were now observed both for singleton probes that matched the upcoming (relevant) target color, and for probes that matched the preceding (now irrelevant) target color. This observation provides clear electrophysiological evidence for the simultaneous activation of two color‐specific target templates. Notably, the relevance of color for the next search episode only affected N2pcs to probes that appeared immediately prior to the arrival of the search display. These probes triggered larger N2pcs when they matched the upcoming target color (rather than the preceding target color). In another recent study from our lab, observers also searched for one of two possible color‐defined targets, but these targets now appeared in a random order, so that their identity was no longer predictable (Grubert & Eimer, 2023). In one condition, these colors were equiprobable (50%). In another condition, they differed in their probability (80% vs. 20%). Probes that matched either of the two target colors triggered N2pc components from about 600–800 ms prior to search display onset, providing further evidence for multiple‐template activation. Notably, these probe N2pcs did not differ from N2pcs triggered by probes in a one‐color task where participants always searched for the same color target. Expectations linked to the a priori probability of a particular target also did not affect the size or time course of these probe N2pcs.

The results of these two studies suggest that during the preparation for two‐color search, both color‐selective templates are activated simultaneously. Because maintaining two target templates at the same time is unlikely to exceed working memory capacity, it may be less demanding to always activate both templates concurrently rather than switching templates, even when only one of them is relevant for the next search episode (ABAB task; Grubert & Eimer, 2020). However, when the number of possible target colors is further increased, limits to the capacity of preparatory target template activation processes may emerge. In the present study, we investigated this possibility by including search tasks in which observers searched for one of three possible color‐defined target objects. Maintaining three color‐specific target templates concurrently might reach (or possibly even exceed) working memory capacity for many observers, and this may result in qualitative differences in the way that these templates are activated during search preparation relative to one‐color or two‐color search.

We employed the same RSPP technique as in our previous work (Grubert & Eimer, 2018, 2020, 2023; see also Dodwell et al., 2024). In Experiment 1, there were three possible color‐defined target objects, but the identity of each target was fully predictable in each trial because the target colors rotated in a constant order between trials (ABCABC). Thus, Experiment 1 was equivalent to our previous ABAB task (Grubert & Eimer, 2020), except that a third target color was added. Color singleton probes were presented rapidly and continuously between search displays, and probes matching each of the three target colors appeared with equal probability and in a random order. The question was whether we would again observe evidence for multiple‐template activation under these circumstances. If all three color templates are activated on any given trial, even though the identity of the target is fully predictable, reliable N2pc components to all color singleton probes should emerge during search preparation. Alternatively, template activation may be fully color‐selective during the predictable three‐color search, so that only the template that matches the upcoming target color is activated at any time. In this case, only these target‐matching probes should trigger N2pc components, whereas no N2pcs should be observed for the two other color singleton probes.

## 2 EXPERIMENT 1

### 2.1 Methods

#### 2.1.1 Participants

Twenty‐two participants were paid at an hourly rate of £10 to participate in Experiment 1. The experiment was approved by the Ethics Committee of the Psychology Department at Durham University and was conducted in accordance with the Declaration of Helsinki. Participants gave informed written consent prior to testing. Four participants were excluded from analysis due to excessive eye movement artifacts (>40% of trials were lost during artifact rejection). The remaining 18 participants were between 19 and 47 years old (mean = 30.0, *SD* = 8.7). Thirteen participants were female and five were male. All participants were right‐handed. They all had normal or corrected‐to‐normal vision and normal color vision (as tested with the Ishihara color vision test; Ishihara, 1972). The sample size of 18 was calculated by means of an a priori power analysis using MorePower 6.0.1 (Campbell & Thompson, 2012) to detect an interaction in a 2 × 7 × 3 factorial repeated‐measures ANOVA (within‐subject factors laterality, probe number, and probe color, see Section 2.2) with an assumed alpha of .05, power of .95, and a large effect size of 0.4 (Cohen's *ƒ*) to replicate partial eta squared values (*η*_p_^2^) of .14, which we measured in a previous RSPP experiment in which participants searched for two alternating target colors (3‐way interaction between laterality × probe number × probe color in Experiment 1 of Grubert & Eimer, 2020, p. 1531).

#### 2.1.2 Stimuli and procedures

**FIGURE 1:**
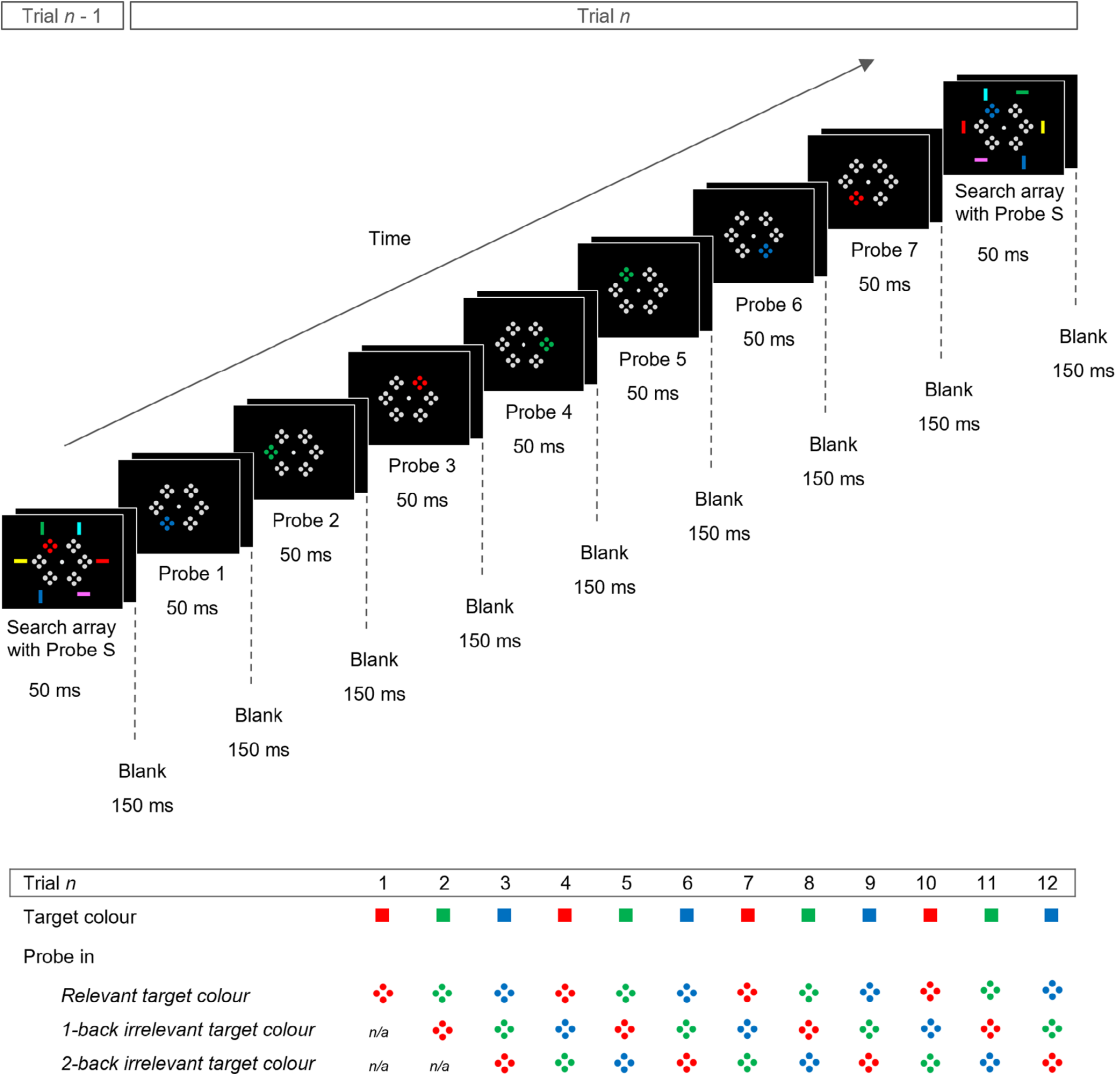
Schematic illustration of the stimuli and presentation times in Experiment 1 (top panel). Search displays contained three target color bars (e.g., red, green, blue), and three non‐target color bars (e.g., yellow, cyan, pink). However, the response‐relevant target color alternated predictably across the 12 trials of each block (e.g., red, green, blue, red, green, blue, etc.; bottom panel). Probe displays were presented every 200 ms in the interval between two search displays (probes 1–7) and simultaneously with a search display. They contained a color singleton that randomly matched the relevant (upcoming) target color, the previous target color (1‐back irrelevant target‐color probes), or the color of the target before the previous trial (2‐back irrelevant target‐color probes).

The experiment was tested in a sound attenuated Faraday cage with dim illumination. The viewing distance from the monitor was approximately 90 cm. Stimuli were presented on a 22‐inch MSI Optix G272 LCD monitor with a 100 Hz refresh rate and a resolution of 1920 × 1080 pixels. Stimulus presentation, timing, and response collection were controlled using PsychoPy (psychophysics software in Python; Peirce et al., 2019) on an LG Pentium PC with Windows 10. All stimuli were presented on a black background. A central gray fixation point was presented throughout the experimental blocks (CIE *x*, *y* color coordinates: .327/.348; 0.2° × 0.2° of visual angle). Each block contained 12 trials with eight stimulus displays that were presented in a continuous serial presentation stream, as illustrated in Figure 1 (top panel). Stimulus displays were presented for 50 ms and were separated by a 150 ms blank interval (200 ms stimulus onset asynchrony). The first seven displays in each trial contained a probe array (probes 1–7), the eighth displays contained both the response‐relevant search array and a probe array. The probes in the eighth display were only presented for the sake of a consistent visual pattern throughout the blocks. They never triggered any N2pcs in our previous work (e.g., Grubert & Eimer, 2018) and will not be analyzed in this study.

Search arrays were presented at an eccentricity of 1.4° from central fixation and contained six vertically (0.2° × 0.6°) or horizontally (0.6° × 0.2°) oriented bars at the 1, 3, 5, 7, 9, and 11 o'clock positions of an imaginary clock face. The orientations of the six bars were selected independently and randomly for each search array. Each bar had a different color. They were red (.610/.321), green (.273/.624), blue (.172/.181), yellow (.435/.490), pink (.483/.246), and cyan (.222/.313). All colors were equiluminant (~11.9 cd/m^2^), and they were allocated randomly to the six bars in each search display. Each participant was assigned three target colors from the set of red, green, blue, and yellow (pink and cyan were dedicated non‐target colors only). The four possible sets of target colors were counterbalanced across participants so that five participants searched for red, green, and blue targets, five other participants searched for red, green, and yellow targets, and always four other participants searched for red, blue, and yellow or green, blue, and yellow targets. However, in each trial, only one of the three colors was response relevant: Target colors alternated sequentially across consecutive trials (e.g., red in trial 1, green in trial 2, blue in trial 3, red in trial 4, etc.). The target color sequence was determined randomly for each participant but remained the same for each participant throughout the experiment. Participants' task was to report the orientation (vertical/horizontal) of the response‐relevant target color bar in each trial by pressing the up/down arrow keys on a standard keyboard. Since search displays always contained all three target colors, participants had to keep track of the target color sequence for themselves. There were no cues indicating the upcoming target color during a block, but participants received a reminder about their target colors and the respective target color sequence in each block break (none of the participants reported forgetting the sequence during a block). The locations of the three target color bars in each search array were determined randomly and independently of each other. The response to key mapping (vertical/horizontal response on arrow up/down key) and the hand‐to‐key mapping (left/right hand on arrow up/down key) was counterbalanced across participants but was kept constant for each participant for the duration of the whole experiment.

Probe arrays contained six items composed of four closely aligned dots, two on the vertical, and two on the horizontal axis (0.1° × 0.1° for each dot, 0.25° × 0.25° for each four‐dot probe item). The probe items were presented at the same 1, 3, 5, 7, 9, and 11 o'clock positions of an imaginary clock face than the search bars, but closer to fixation (at an eccentricity of 0.5°). Five of the six probe items were uniformly gray, the sixth item was a target color singleton that randomly matched any of the three respective target colors (as assigned to each participant). Probes that matched the color of the upcoming search target were *relevant target‐color probes*, probes that matched the target color of the previous trial were *1‐back irrelevant target‐color probes*, and probes that matched the target color of the target before the previous trial were *2‐back irrelevant target‐color probes* (Figure 1, bottom panel). The singleton locations were selected randomly and independently in each probe array with the following two restrictions. Successive probes were equally likely to appear on same or opposite display sides to avoid any hemispheric imbalance in the baseline activity preceding each probe, and immediate location repetitions between probe displays were not allowed to avoid color masking effects (note that the location variability was therefore reduced in same side as compared to opposite side probes). Participants were informed that probe displays were never response‐relevant and could be ignored.

Experiment 1 contained 55 blocks of 12 trials. Blocks were kept as short as possible and participants were instructed not to blink during the blocks, if possible. The twelfth search display in each block was followed by seven additional probe displays to keep stimulus conditions during the post‐target response interval identical across all trials in a block. Each block thus contained 12 search displays and 91 probe displays (13 for each probe number 1–7). Before the experiment proper, participants practised the task until they felt comfortable with it (usually after two to four blocks). These practise data were not recorded.

#### 2.1.3 EEG recording and data analyses

EEG was DC‐recorded from 25 scalp sites (at standard positions of the extended 10/20 system; EasyCap, Brain Products), sampled at 500 Hz (BrainAmp DC amplifier, Brain Products), and digitally low‐pass filtered at 40 Hz (no other filters were applied after data acquisition). Impedances were kept below 5kΩ. The left earlobe served as the online reference during data acquisition. Offline, all channels were re‐references to linked earlobes. EEG data processing was conducted with the BrainVision Analyzer software (Brain Products GmbH, Gilching, Germany). EEG epochs were locked to the onsets of the probes (probes 1–7) and the search displays and included a 100 ms pre‐stimulus baseline and a 400 ms post‐stimulus ERP time window. Data from the first and last seven probe displays in each block were excluded from analysis. Probes that were presented prior to search displays with anticipatory (<200 ms), very slow (>1500 ms), incorrect, or missing responses were also excluded from analysis. Epochs that were contaminated with artifacts were also excluded from analysis. Artifacts were eye movements (±30 μV in the bipolar HEOG channel), blinks (±60 μV at Fpz), and other muscular activity (±80 μV in all channels). Artifact rejection resulted in an exclusion of 8.7% of all epochs (*SD* = 7.2%; ranging between 0.8% and 25.3% across participants). The remaining epochs were averaged separately for each probe number (probes 1–7) for relevant, 1‐back irrelevant, and 2‐back irrelevant target‐color singletons in the left versus right hemifield (mean number of epochs for each average = 87; *SD* = 10; ranging between 56 and 97 epochs across participants). Separate averages were also computed for search displays with a target in the left or right hemifield (M = 285 per average; *SD* = 19; ranging between 250 and 319 epochs across participants).

N2pc components to probes were quantified based on ERP mean amplitudes obtained at lateral posterior electrodes PO7 and PO8, contralateral and ipsilateral to the side of a probe, within an 80 ms time window starting at 190 ms after the respective probe display onset. As in our previous work using analogous RSPP procedures (Grubert & Eimer, 2018), the start of this time window was determined by measuring the point in time (rounded to the nearest 10) when the ascending flank of the averaged probe N2pc (pooled across all relevant target‐color probes in Experiment 1) reached 50% of the peak amplitude (at −0.13 μV). Target N2pcs in the search displays were computed within the same 190–270 ms post‐stimulus time window for consistency. Target N2pc onset latencies were substantiated by means of jackknife‐based procedures (Miller et al., 1998). Eighteen grand‐average difference waves (contralateral minus ipsilateral ERPs at PO7/8) were computed, each excluding one different participant from the original sample. N2pc onset latencies were defined as the point in time when each subsample difference wave reached an absolute onset criterion of −0.7 μV (50% of the peak amplitude of the target N2pc in Experiment 1). All *t* tests on jackknifed N2pc onset latencies were power‐corrected as suggested by Miller et al. (1998) and are denoted with *t*_c_. All *t* tests reported are two‐tailed. Effect sizes are reported in terms of Cohen's *d* (Cohen, 1988), with a confidence interval of 95%, for *t* tests, and partial eta squared (*η*_p_^2^) for *F* tests and power‐corrected *t*_c_‐tests (*η*_pc_^2^).

### 2.2 Results

#### 2.2.1 Behavioral results

Trials with anticipatory (<200 ms) or exceedingly slow (>1500 ms) responses were excluded from analysis (0.5% of all trials). The mean reaction time (RT) in correct trials was 679 ms and the mean error rate was 8.3%.

#### 2.2.2 N2pc components triggered in the probe displays

**FIGURE 2:**
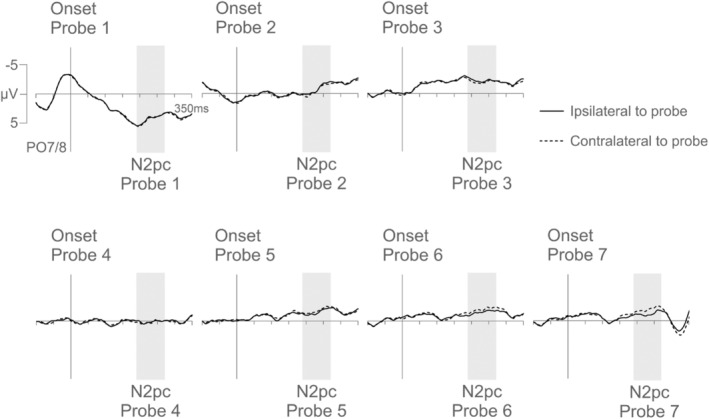
Grand‐averaged ERPs triggered at electrode sites PO7/8 contralateral and ipsilateral to relevant target‐color singleton in the seven probe displays presented between consecutive search displays of Experiment 1. Probe 1 is the first probe to follow the previous search display and probe 7 is the probe to immediately precede the next search display. Shaded areas mark N2pc time windows (190–270 ms after the onset of each individual probe).

**FIGURE 3:**
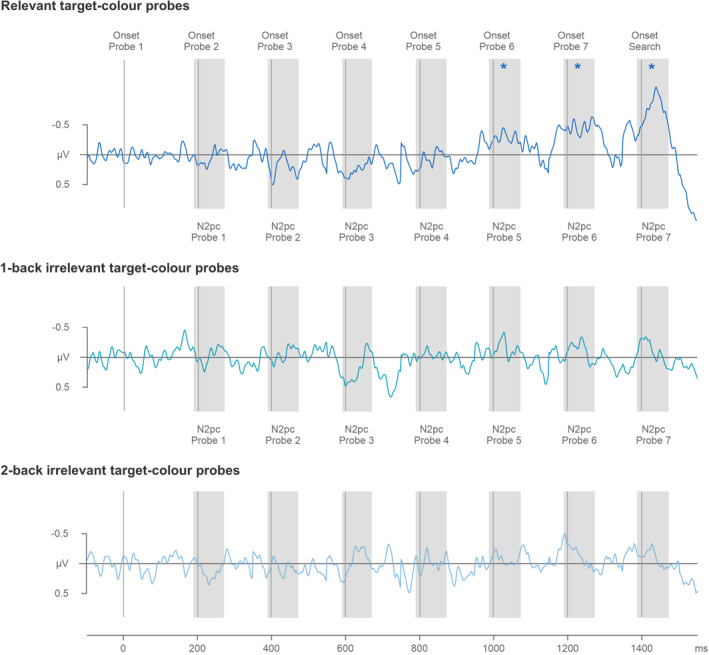
N2pc difference waveforms obtained by subtracting ipsilateral from contralateral ERPs for relevant target‐color probes (top panel), 1‐back irrelevant target‐color probes (middle panel), and 2‐back irrelevant target‐color probes (bottom panel) in Experiment 1. Here, difference waves for the seven probes (probes 1–7) are illustrated in a temporally continuous fashion, but the seven individual probe N2pcs were extracted independently of each other from the raw signal. Probe onsets are indicated by vertical lines, and probe N2pc time windows by shaded areas (190–270 ms after the onset of each individual probe). Statistically reliable probe N2pcs are marked by asterisks.

To determine the time course of template activation in preparation for search, N2pc components triggered in each of the seven successive probes (probes 1–7) were measured by computing ERPs at posterior sites PO7/8, contralateral and ipsilateral to the side of a probe, separately for probes that matched the upcoming target‐color (relevant target‐color probes), the previous target color (1‐back irrelevant target‐color probes), and the target color that was relevant before the previous target color (2‐back irrelevant target‐color probes). The ERPs for relevant target‐color probes 1–7 are illustrated in Figure 2 (the corresponding ERPs to 1‐back and 2‐back irrelevant target‐color probes are included in the Materials S1). Probe N2pc difference waves, obtained by subtracting ipsi‐ from contralateral ERPs at PO7/8 for each individual probe, are shown in Figure 3. To make the time course of the successive probe N2pcs easier to see, Figure 3 was designed to show the N2pc difference waves for probes 1–7 in a temporally continuous fashion, separately for relevant (top panel), 1‐back irrelevant (middle panel), and 2‐back irrelevant (bottom panel) target‐color probes. Note that N2pc components were extracted individually for each probe (probes 1–7) and that Figure 3 simply illustrates these probe N2pcs in a successive fashion. Figure 3 starts with the activity triggered in response to probe 1 (100 ms prior to 350 ms after the onset of probe 1) which was the first probe presented directly after a previous search display. For the subsequent probes (probes 2–7), 200 ms intervals (150–350 ms after the onset of each respective probe) are shown sequentially with interpolated data points between adjacent intervals. The onset of each probe is marked with a vertical line, and the N2pc time window for each probe (190–270 ms post‐stimulus) is shaded in gray. As probes appeared every 200 ms, each individual probe was therefore presented within the N2pc time interval of its immediately preceding probe. As can be seen from Figure 3, relevant target‐color probes triggered N2pc components in the second half of the preparation period before search onset with the N2pc for probe 7, immediately preceding the next search display, being the largest. Relevant target‐color probes that were presented earlier in the trial did not trigger any N2pcs. This N2pc distribution mirrors our previous RSPP findings (e.g., Grubert & Eimer, 2018) and demonstrates that attentional templates are activated in a transient fashion during preparation for search. In contrast to the N2pc pattern triggered by relevant target‐color probes, none of the probes that matched any of the previous target colors (1‐back and 2‐back irrelevant target‐color probes) seemed to trigger any substantial N2pc components.

Statistical analyses confirmed these informal observations. ERP mean amplitudes measured at PO7/8 in the 190–270 ms post probe time windows were fed into a repeated‐measures omnibus ANOVA with the factors probe color (relevant, 1‐back irrelevant, and 2‐back irrelevant target‐color probe), probe number (probes 1–7), and Laterality (electrode contralateral and ipsilateral to the hemifield of a probe). There was no main effect of Laterality, *F*(1,17) = 1.9, *p* = .186, *η*_p_^2^ = .10, but a significant interaction between laterality and probe number, *F*(6,102) = 3.6, *p* = .003, *η*_p_^2^ = .17, confirming that some of the probes triggered N2pc components, while others did not. There was also an interaction between laterality and probe color, *F*(2,34) = 3.3, *p* = .050, *η*_p_^2^ = .16, and a significant three‐way interaction, *F*(12,204) = 1.8, *p* = .046, *η*_p_^2^ = .10. This suggests that the temporal pattern of probe N2pcs not only differed across consecutive probes but was also different for relevant and irrelevant target‐color probes.

The differences between N2pcs triggered in response to relevant, 1‐back irrelevant, and 2‐back irrelevant target‐color probes were followed up with three repeated‐measures ANOVAs with the factors probe number (probes 1–7) and laterality (contralateral versus ipsilateral activity). For relevant target‐color probes, the ANOVA produced a main effect of Laterality, *F*(1,17) = 6.0, *p* = .025, *η*_p_^2^ = .26, and a significant interaction between laterality and probe number, *F*(6,102) = 5.3, *p* < .001, *η*_p_^2^ = .24, confirming that probe N2pc amplitudes differed across the preparation period. Follow‐up *t* tests, comparing ipsi‐and contralateral activity for each of the seven consecutive probes separately, revealed that probe 5 (−0.2 μV), *t*(17) = 2.3, *p* = .044, *d* = 0.11, probe 6 (−0.3 μV), *t*(17) = 4.8, *p* < .001, *d* = 0.17, and probe 7 (−0.6 μV), *t*(17) = 3.9, *p* < .001, *d* = 0.39, triggered reliable N2pc components. In contrast, no N2pcs were triggered in response to probes 1–4, all *t*(17) < 1, *p* > .661, *d* < 0.01. The same ANOVAs for 1‐back and 2‐back irrelevant target‐color probes did not produce any main effects of laterality, both *F*(1,17) < 1, *p >* .768, *η*_p_^2^ < .05, and also no significant probe number × Laterality interactions, both *F*(1,17) < 1.1, *p >* .412, *η*_p_^2^ < .06, demonstrating that irrelevant target‐color probes never triggered any N2pc components.

#### 2.2.3 N2pc components triggered in the search displays

**FIGURE 4:**
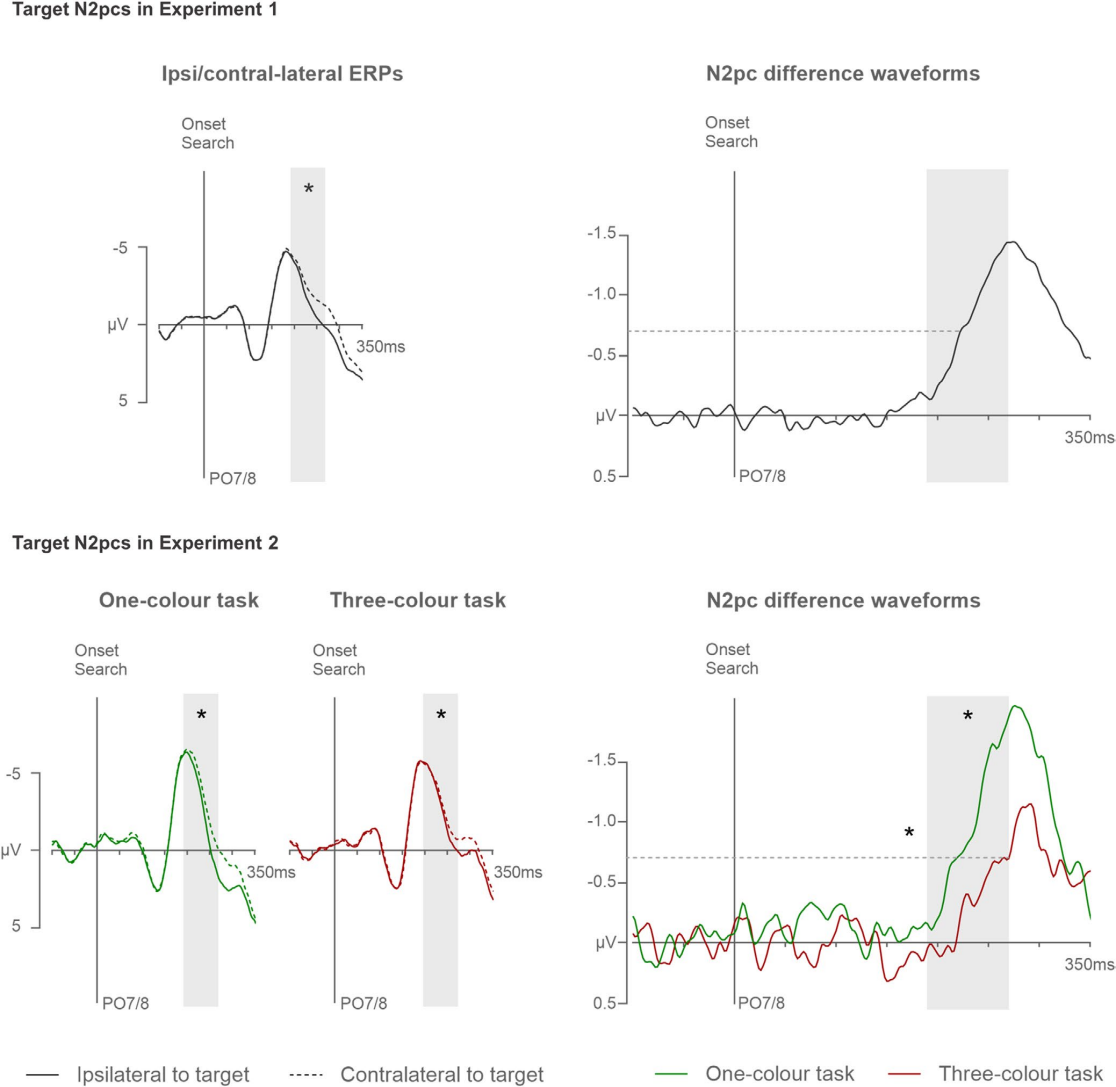
Grand‐averaged ERPs triggered at electrode sites PO7/8 contralateral and ipsilateral to the target side in the three‐color search displays of Experiment 1 (top left panel) and the one‐ and three‐color search displays of Experiment 2 (bottom left panels). The corresponding contralateral‐ipsilateral N2pc difference waveforms are shown in the top and bottom right panels, respectively. Shaded areas indicate N2pc time windows (190–270 ms after search display onset). Asterisks in the ERP panels (left) indicate significant N2pcs. Asterisks in the difference wave panels (right) represent significant task differences in mean amplitudes and onset latencies (measured at −0.7 μV, as indicated by the dashed horizontal lines).

Target N2pcs were substantiated at PO7/8 ipsilateral and contralateral to the side of the target in the 190–270 ms time window after search display onset. These ERPs, together with the respective N2pc difference waves are shown in Figure 4 (top panel). A *t* test comparing ipsi‐ and contralateral activity confirmed that target N2pc mean amplitudes were reliable (−0.8 μV), *t*(17) = 5.3, *p* < .001, *d* > 0.22. The onset latency of the target N2pc was 224 ms.

### 2.3 Discussion of experiment 1

The pattern of probe N2pc results obtained in Experiment 1 was clear‐cut. Reliable N2pcs were only triggered by color singleton probes that matched the known color of the upcoming target, but not by probes that matched the colors of the other two possible targets. Probes in the currently relevant color triggered reliable N2pcs when they were presented during the 600 ms prior to the next search display (probes 5–7), but not when they appeared earlier in the preparation period (probes 1–4). In line with previous observations (e.g., Grubert & Eimer, 2018), this demonstrates that target‐color templates were only active in temporal proximity to an upcoming search episode, rather than throughout the entire preparation period. In contrast, color singleton probes that matched the colors of either of the two previous targets failed to trigger any N2pcs at all, suggesting that the corresponding color templates remained inactive.

These results are qualitatively different from the results of a previous study (Grubert & Eimer, 2020) that used identical procedures except, that participants searched for one of two rather than three predictably alternating targets (ABAB versus ABCABC). Whereas probe N2pcs revealed the concurrent activation of both target color templates in this earlier study, the addition of a third color target in Experiment 1 resulted in color‐selective preparation that was limited to the upcoming target color. This change in search preparation might have been a strategic choice of the participants to reduce the working memory load and focus on the one known (as opposed to three possible) target color(s). Alternatively, it might also reflect capacity limitations, with the number of target templates that can be maintained simultaneously being limited to two. If this was the case, the fact that target colors were fully predictable in Experiment 1 would make the adoption of a single‐template strategy the most adaptive choice. Experiment 2 was conducted to test this hypothesis directly and to substantiate potential differences in template activation patterns during three color search in which all color templates were equally relevant for the upcoming search.

## 3 EXPERIMENT 2

If maximally two preparatory target templates can be activated in parallel, this should have adverse effects on attentional guidance in three‐color search tasks where the identity of each color target is no longer fully predictable (as in Experiment 1) but instead varies randomly across trials. Template activation under such different task demands was tested in Experiment 2, which included two search tasks. There was a three‐color task that was identical to the ABC task of Experiment 1, with two exceptions. First, and most importantly, the three possible target colors now appeared in random order across search displays, so that each target color was equally likely to be presented in any given search display. Second, the color singleton probes now either matched any of the three possible target colors or a distractor color that also appeared in the search displays. Distractor‐color probes were included in Experiment 2 to rule out the possibility that N2pcs to target‐color probes might at least in part reflect salience‐driven attentional capture by singleton probes that are unrelated to any target template activation. In previous studies (Grubert & Eimer, 2018, 2023), such distractor‐color probes failed to trigger any N2pcs. They were still included in Experiment 2 because of the possibility that salience‐driven attentional capture might emerge in search tasks where the number of possible target representations exceeds the capacity limits for multiple‐template activation.

If no more than two color templates can be activated simultaneously during search preparation, participants will not be able to fully prepare for all potential target colors when three different color targets vary unpredictably across trials. In such a situation, they may abandon any preparatory template activation altogether, which should be reflected in the absence of any N2pc components in response to target‐color singleton probes. Alternatively, they may randomly activate two target‐color templates on each trial. This should result in an overall reduction of probe N2pc amplitudes, as one third of all target‐color probes will not match either of the active templates and thus not attract attention and trigger an N2pc. To assess whether target‐color probes are indeed attenuated in the three‐color task, Experiment 2 also included a one‐color task where participants always searched for the same color‐defined target object. Again, color singleton probes either matched the color of this target or appeared in a task‐irrelevant distractor color. In this one‐color task, the corresponding attentional template should always be activated during search preparation, resulting in full‐size N2pc components in response to target‐color cues. In contrast, if search preparation is limited to two (or one) target colors in the three‐color task, this should result in reliably reduced probe N2pc components in this task relative to the one‐color task. Finally, it might also be possible that three target‐color templates are activated in parallel, but that the color representations are less precise for three as opposed to two concurrently activated colors (in line with resource models of working memory, e.g., Bays & Husain, 2008). This might also lead to attenuated target‐color probe N2pcs in the three‐color as compared to the one‐color task. But in this scenario, we would also expect target‐similar distractor probes (e.g., pink probes during the search for red) to capture attention (e.g., Kerzel, 2019). Distractor‐color probes should therefore trigger N2pc components in the three‐color but not the one‐color task in which target representations should be completely precise and distractor‐color probes should be fully ignored.

In addition to comparing probe N2pcs between the one‐color and three‐color tasks, we also compared the N2pc components triggered by target objects in search displays between these two tasks. Previous studies (e.g., Berggren & Eimer, 2019; Grubert & Eimer, 2016, 2023; Ort et al., 2019) have consistently found that target N2pcs are attenuated and delayed during two‐color as compared to one‐color search, suggesting that the guidance of attentional selectivity is less efficient when two color templates are active (see also Ort & Olivers, 2020). If only two target templates can be activated concurrently, this difference might be even more pronounced when contrasting one‐color and three‐color search.

### 3.1 Methods

#### 3.1.1 Participants

Twenty‐three new participants were paid at an hourly rate of £10 to participate in Experiment 2. Participant procedures were identical to Experiment 1. Four participants were excluded due to excessive eye movement activity (>40% trials lost during artifact rejection) and one additional participant was excluded because they did not finish the task. The remaining 18 participants were aged between 20 and 25 years (mean = 29.5, *SD* = 10.3). Eleven participants were female and seven were male, all of them were right‐handed, and had normal or corrected‐to‐normal vision and normal color vision (as tested with Ishihara, 1972).

#### 3.1.2 Stimuli and procedures

All experimental procedures were identical to Experiment 1 with some exceptions that are explained below. In Experiment 2, the three target colors assigned to each participant were now presented randomly. Because the response‐relevant target color now changed unpredictably between trials, participants had to activate three color templates in parallel to enable target detection. Nine of the participants searched for red, green, and blue targets, while pink, yellow, and cyan were the designated distractor colors, and vice versa for the other nine participants. This color assignment ensured that target colors were separable in color space so that participants could not adopt a relative color template for guidance (Becker, 2010). Each search display always contained one target color bar (e.g., red), three distractor color bars (e.g., pink, yellow, cyan), and two dedicated non‐target color bars (gray and brown). The six different colors were allocated randomly to the six bars in each search display. Experiment 2 was run on Matlab using the Cogent 2000 Toolbox and was tested on a different monitor than Experiment 1 (17‐inch Samsung wide Syncmaster 753S CRT; 1280 × 1024‐pixel resolution; 100‐Hz refresh rate). The color values, therefore, slightly differed from Experiment 1: red (.609/.327), green (.296/.581), blue (.174/.149), pink (.216/.110), yellow (.389/.512), cyan (.227/.376), gray (.287/.312), and brown (.540/.400). All colors were equiluminant (∼10.9 cd/m2). Half of all probes in Experiment 2 were *target‐color probes* and contained one of the three target colors (e.g., red, green, blue). The remaining probes were *distractor‐color probes* shown in one of the three distractor colors (e.g., pink, yellow, cyan). The exact color of the probe singleton, from either the target or distractor color set, was chosen randomly and independently in each probe display. In addition to the *three‐color search*, we also tested a one‐color version of this task in Experiment 2. In the *one‐color task*, each participant searched for one of the three target colors they were assigned to in the three‐color task (e.g., red). The other two colors of the respective target color set never appeared in the search or probe displays (e.g., green, blue), so that previous target colors would never become distractors. Half of the color singletons in the probe displays were shown in the designated target color (e.g., red) and the other half in any of the three colors from the respective distractor color set (e.g., pink, yellow, cyan). The one‐color and three‐color search tasks were tested in 30 separate blocks each, with 12 trials per block.

#### 3.1.3 EEG recording and data analyses

All EEG procedures were identical to Experiment 1. During artifact rejection, 10.1% of all segments in the one‐color task (*SD* = 8.5%; ranging between 0.3% and 25.0% across participants) and 9.2% in the three‐color task (*SD* = 8.4%; ranging between 0.5% and 29.4% across participants) were excluded from analysis in Experiment 2. Averaged ERP waveforms were computed for probes 1–7 in the left or right hemifield, separately for target and distractor color probes in the one‐color (mean number of epochs for each average = 77; *SD* = 6; ranging between 59 and 83 epochs across participants) and three‐color task (M = 72 per average; *SD* = 8; ranging between 58 and 82 epochs across participants). Separate averages were computed for left‐ and right‐side targets in the search displays in the one‐color (M = 152 per average; *SD* = 23; ranging between 104 and 173 epochs across participants) and three‐color task (M = 147 per average; *SD* = 22; ranging between 98 and 174 epochs across participants).

All data analysis procedures were identical to Experiment 1.

### 3.2 Results

#### 3.2.1 Behavioral results

Trials with anticipatory or slow responses were excluded from the analysis (0.2% of all trials). RTs in correct trials were faster and error rates were lower in the one‐color (610 ms; 4.4%) as compared to the three‐color task (731 ms; 13.0%), both *t*(17) > 4.7, *p* < .001, *d* = 0.57.

#### 3.2.2 N2pc components triggered in the probe displays

**FIGURE 5:**
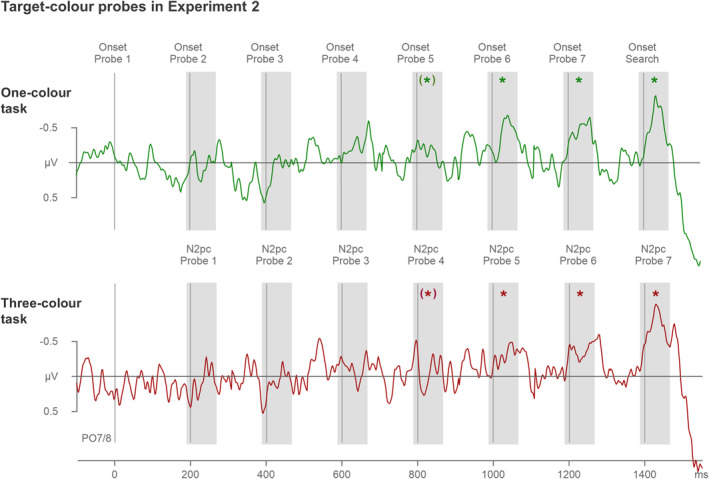
N2pc difference waveforms obtained by subtracting ipsilateral from contralateral ERPs triggered by the target‐color probes in the one‐color (top panel) and three‐color tasks (bottom panel) of Experiment 2. Difference waves triggered by individual probes are shown in the same continuous fashion as in Figure 3. Probe onsets are indicated by vertical lines, and probe N2pc time windows by shaded areas (190–270 ms after the onset of each individual probe). Statistically reliable probe N2pcs are marked by asterisks. Note that probe 4 N2pcs were reliable when power was combined across the two tasks (repeated‐measures ANOVA), but that individual t tests for probe 4 N2pcs failed to reach significance in both tasks.

**FIGURE 6:**
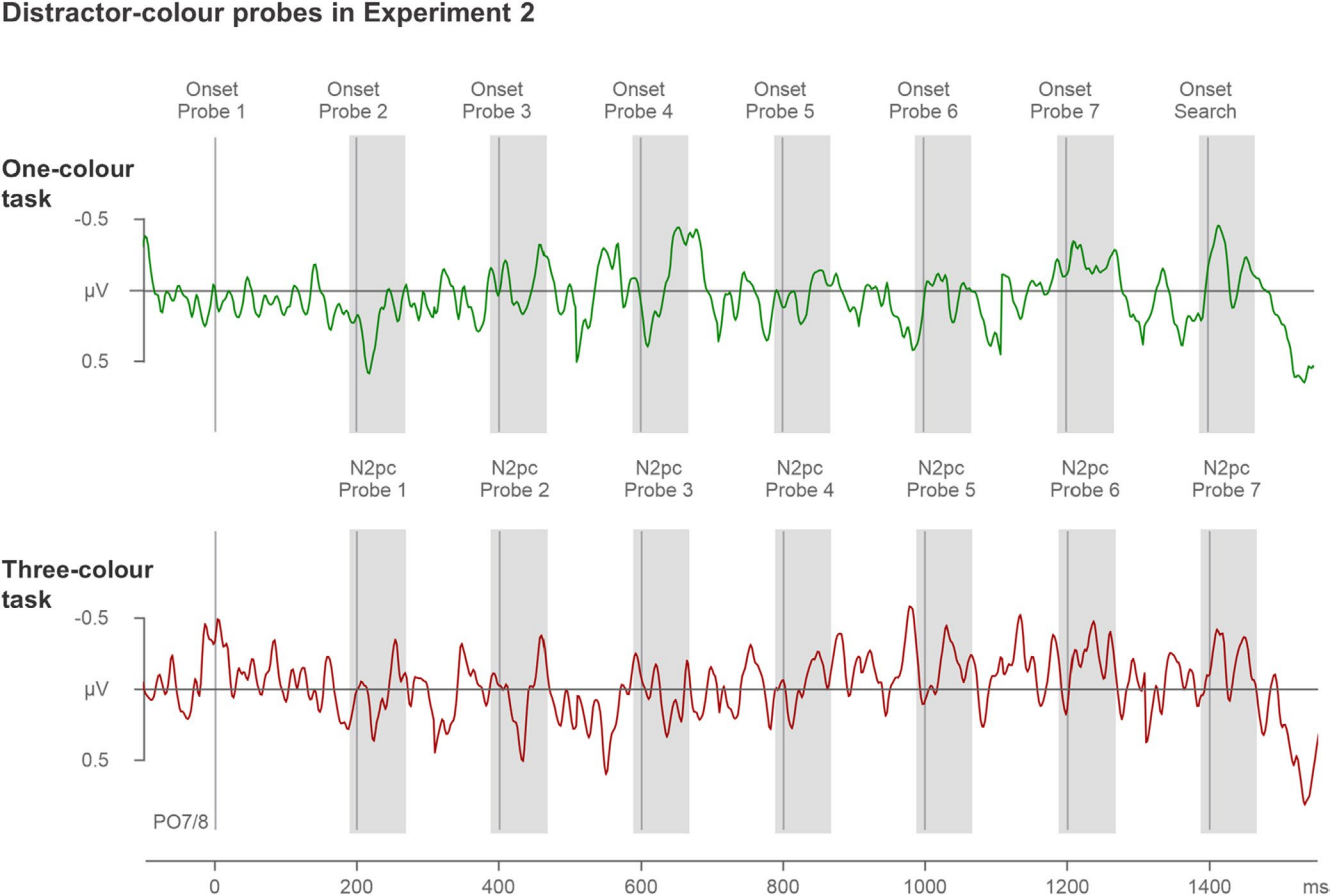
N2pc difference waveforms obtained by subtracting ipsilateral from contralateral ERPs triggered by the distractor‐color probes in the one‐color (top panel) and three‐color tasks (bottom panel) of Experiment 2. Difference waves triggered by individual probes are shown in the same continuous fashion as in Figures 3 and 5. Probe onsets are indicated by vertical lines, and probe N2pc time windows by shaded areas (190–270 ms after the onset of each individual probe).

The temporally continuous N2pc difference waves (obtained by subtracting ipsi‐ from contralateral ERPs at PO7/8) can be seen in Figures 5 and 6. The difference waves are shown separately for target‐color (Figure 5) and distractor‐color probes (Figure 6) in the one‐color (top panels) and three‐color search tasks (bottom panels), respectively. The corresponding ipsi/contra lateral ERPs can be found in Materials S1. The temporal pattern of target‐color probe N2pcs mirrored the N2pc pattern observed for relevant target color probes in Experiment 1, with pronounced N2pcs emerging in the later phase during search preparation. However, distractor‐color probes did not seem to trigger any N2pc components in either the one‐color or the three‐color search task.

ERP mean amplitudes measured at PO7/8 in the 190–270 ms post‐probe time windows were subjected to a repeated‐measures omnibus ANOVA with the factors Task (one‐color, and three‐color search), probe color (target‐color, and distractor‐color probe), probe number (probes 1–7), and Laterality (electrode contralateral and ipsilateral to the hemifield of a probe). The ANOVA revealed a main effect of Laterality, *F*(1,17) = 22.2, *p <* .001, *η*_p_^2^ = .57, and two significant interactions between laterality and probe number, *F*(6,102) = 6.3, *p <* .001, *η*_p_^2^ = .27, and laterality and probe color, *F*(1,17) = 24.1, *p <* .001, *η*_p_^2^ = .59. Interestingly, none of the interactions involving the factor task reached significance, all *F* < 1, *p >* .688, *η*_p_^2^ < .04.

The effects of probe color were followed up by means of two repeated‐measures ANOVAs with the factors task (one‐color, three‐color), probe number (probes 1–7), and Laterality (contralateral, ipsilateral activity), separately for target‐ and distractor‐color probes. The ANOVA on target‐color probe N2pcs uncovered a main effect of Laterality, *F*(1,17) = 43.8, *p* < .001, *η*_p_^2^ = .72, and a significant interaction between Laterality and probe number, *F*(6,102) = 7.2, *p* < .001, *η*_p_^2^ = .30, indicating that probe N2pc amplitudes differed across the preparation period. The absence of any interaction involving the factor Task, all *F* < 1, *p* > .992, *η*_p_^2^ < .02, suggests that the pattern of probe N2pcs across the preparation period was identical in the one‐color and three‐color tasks. Follow‐up ANOVAs with the factors Task (one‐color, three‐color) and Laterality (contralateral, ipsilateral) revealed main effects of Laterality for probe 4 (−0.2 μV), *F*(1,17) = 4.8, *p* = .042, *η*_p_^2^ = .22,^1^ probe 5 (−0.3 μV), *F*(1,17) = 15.5, *p* = .001, *η*_p_^2^ = .48, probe 6 (−0.4 μV), *F*(1,17) = 31.6, *p* < .001, *η*_p_^2^ = .65, and probe 7 (−0.6 μV), *F*(1,17) = 34.1, *p* < .001, *η*_p_^2^ = .67. Probes 1–3 did not produce any significant N2pcs, all *F*(1,17) < 3.3, *p* > .089, *η*_p_^2^ < .16. None of these ANOVAs for individual probes produced a reliable interaction with Task, all *F*(1,17) < 1, *p* > .482, *η*_p_^2^ < .03, indicating that not only the pattern of probe N2pcs, but also the size of the N2pcs triggered by the probes at different temporal positions prior to search were identical in the one‐color and three‐color task.

The ANOVA on distractor‐color probe N2pcs did not produce any main effects of Laterality, *F*(1,17) = 2.4, *p* = .140, *η*_p_^2^ = .12, or any significant interactions involving the factor Laterality, all *F* < 1, *p* > .618, *η*_p_^*2*^ < .04. In other words, none of the distractor‐color probes produced any N2pcs, either in the one‐color or the three‐color task.

#### 3.2.3 N2pc components triggered in the search displays

Target ERPs and N2pc difference waves, measured at PO7/8 ipsilateral and contralateral to the side of a target in the 190–270 ms time window after search display onset in the one‐color and three‐color tasks, are shown in Figure 4 (bottom panel). A repeated‐measures ANOVA with the factors Task (one‐color, three‐color) and Laterality (contralateral, ipsilateral), showed a main effect of Laterality, *F*(1,17) = 56.2, *p* < .001, *η*_p_^2^ = .77, and a significant Task × Laterality interaction, *F*(1,17) = 5.9, *p* = .027, *η*_p_^2^ = .26, indicating that targets in both search tasks triggered solid N2pc components, which were significantly larger in the one‐color (−1.0 μV) as compared to the three‐color task (−0.7 μV).^2^ Target N2pcs in the one‐color task (227 ms) were also significantly faster than in the three‐color task (271 ms), *t*_c_(17) = 5.0, *p* < .001, *η*_pc_^2^ = .62.

### 3.3 Discussion of experiment 2

In Experiment 1 with alternating target colors, we observed strategic color‐selective preparation that was limited to one rather than three target colors. In the three‐color task of Experiment 2, one of three possible color‐defined targets appeared randomly and unpredictably on each trial. In such a context, optimal search preparation will involve the concurrent activation of all three target color templates. It is conceivable that the color‐selective preparation observed in Experiment 1 was not strategic, but an effect of capacity limitations on the number of search templates that can be maintained simultaneously. In this case, it should not have been possible to concurrently activate three color templates in Experiment 2. However, the probe N2pc results obtained in the three‐color task of Experiment 2 do not provide any evidence for the existence of such a rigid capacity limitation. Target‐color probes triggered reliable N2pc components from about 600 ms prior to search display onset, demonstrating that color templates were indeed activated during search preparation. The fact that distractor‐color singleton probes did not trigger any N2pcs showed that the presence of target‐color probe N2pcs was not in any way related to salience‐driven attentional capture.

Most notably, there was no difference in the time points when probe N2pcs emerged during the preparation period, or in the size of these N2pcs, between the three‐color and one‐color tasks. This is an important observation because it shows that preparatory template activation processes were equivalent regardless of whether the search task required the activation of one constant or three different color templates. In order words, it strongly suggests that there is no rigid capacity limitation for the activation of multiple attentional templates and that at least three templates can be activated in parallel without apparent costs. Further evidence for the absence of any capacity limitations during preparation for the three‐color search comes from the observation that distractor‐color probes were fully ignored and did not trigger any N2pcs, neither in the one‐ or the three‐color task. This suggests that the precision of the target color representations held in working memory did not suffer in the three‐color as compared to the one‐color task.

In contrast to the apparent absence of any capacity limits of template activation during the preparation period, clear differences between the one‐color and three‐color tasks emerged once a search display had been presented. There were pronounced behavioral costs associated with multiple‐color search, as RTs were delayed by more than 100 ms, and error rates were three times higher in the three‐color as compared to the one‐color task. This was mirrored by N2pc components triggered in response to search targets, which were attenuated and delayed during the three‐color search.^3^ These behavioral and electrophysiological search costs are in line with previous studies contrasting one‐ and two‐color search tasks (e.g., Grubert & Eimer, 2016; Irons et al., 2012), and demonstrate again that attentional guidance and/or subsequent processes involved in search target selection and identification operate less efficiently when the identity of this target is uncertain. The factors that may be responsible for the remarkable contrast between the absence of any multiple‐target costs during search preparation and the presence of large costs during the search episode itself will be considered in the General Discussion.

## 4 GENERAL DISCUSSION

The goal of this study was to employ ERP markers of target template activation during search preparation to investigate capacity limitations associated with activating multiple templates simultaneously, and the costs that arise because of such limitations. Previous work has shown that at least two color‐specific target templates can be activated at the same time (e.g., Grubert & Eimer, 2016, 2023; Irons et al., 2012). To study whether this number represents an upper capacity limit, we employed search tasks where participants searched for one of three possible color‐defined targets, and measured N2pc components elicited by target‐matching and target‐non‐matching color singleton probes that appeared in rapid succession during the interval between two search displays.

In Experiment 1, where the three different targets appeared in a fixed order (ABCABC), so that target identity was fully predictable, reliable N2pc components were elicited only by singleton probes that matched the color of the upcoming target, but not by probes that matched either of the other two target colors. This demonstrates that search preparation was color‐selective and restricted to a single attentional template in this experiment. In contrast, when task demands changed so that the three color‐defined targets were randomly intermixed and target identity was thus no longer predictable (Experiment 2), all three target‐matching singleton probes triggered N2pc components. This indicates that three color templates were activated in parallel during the unpredictable three‐color search. The fact that these N2pc components were equivalent in size to the N2pcs triggered by target‐matching probes in a one‐color task where only a single attentional template was task‐relevant suggests that multiple target‐color templates can be activated without apparent costs relative to single‐template search (but see below for some caveats). Overall, these results imply that template activation during the three‐color search can be limited to a single template when the target identity is predictable. However, this is not the result of rigid capacity limitations, as it is possible to maintain at least three different preparatory color templates simultaneously when this is required because three different color targets are equally likely to appear in any given search display (see also Kerzel & Grubert, 2022, for behavioral support for this conclusion).

The observation that only the color template that was relevant for the next search episode was activated in Experiment 1 during fully predictable three‐color search (ABCABC) raises the question why two color templates were activated in our previous study (Grubert & Eimer, 2020) which was identical to this experiment, with the only difference that two (ABAB), as opposed to three, target colors were employed in the earlier study. There are several possible reasons for this difference. First, given that the cognitive load associated with maintaining multiple attentional templates in working memory increases with each template that is added, participants should have a stronger incentive to adopt a single‐template strategy during the predictable three‐color search than during a two‐color search. Second, during the three‐color search, displays with a particular color target were always followed by two search displays with different color targets. During the two color‐search, the same color target appeared in every second display. The longer interval between search displays with the same relevant target color in the three‐color task may have further encouraged the preparation of a single‐color template in the current Experiment 1 (see Grubert et al., 2024, and Lien et al., 2010, for electrophysiological and behavioral evidence, respectively, of single template activation in AABB designs when the temporal interval between relevant templates is increased as compared to ABAB designs). Both factors (increased cognitive load and longer gaps between target repetitions) may have combined to produce the difference in template activation strategies between predictable two‐color and three‐color searches.

The electrophysiological and behavioral results of Experiment 2 present an interesting conundrum. While the pattern of probe‐induced N2pc components suggests equally strong target template activation during one‐color and three‐color search, behavioral performance and N2pc components and electrophysiological effects in response to search displays suggest clear capacity limitation for three‐color search. This dissociation is consistent with previous behavioral (Kerzel & Grubert, 2022) and ERP studies (Grubert et al., 2016; Grubert & Eimer, 2023; Ort et al., 2019), which also found costs for multiple‐color versus single‐color search primarily for target selection but not during search preparation (see also Ort & Olivers, 2020, for further discussion).

One obvious factor that contributes to costs associated with multiple‐color as compared to single‐color search is the fact that target colors are uncertain in the former case but fully predictable in the latter case. Multiple‐color costs may also be the result of the existence of inhibitory links between multiple simultaneously active target templates (e.g., Grubert et al., 2016; Kerzel & Grubert, 2022; Ort et al., 2019). Between‐template suppression during two‐color or three‐color search will result in lower overall template activation levels as compared to one‐color search, and this should result in less efficient template‐guided target selection, as reflected by behavioral and electrophysiological costs for multiple‐color search observed here and in prior studies (see Kerzel & Grubert, 2022, for specific model predictions). But if there is mutual inhibition between concurrently active templates, why is this not also reflected by a corresponding attenuation of N2pcs to template‐matching probes presented during the preparation for multiple‐color as compared to single‐color search? To answer this question, it is important to stress that probe N2pc components do not reflect search template activation levels directly, but instead the allocation of attention to a color singleton probe that is guided by a matching template. Such interactions between a search template and a template‐matching visual object may trigger an additional transient increase in the activation of this particular template (see also Moore & Weissman, 2010, 2014, for similar suggestions), resulting in similar probe N2pc amplitudes during one‐color and three‐color search.

**FIGURE 7:**
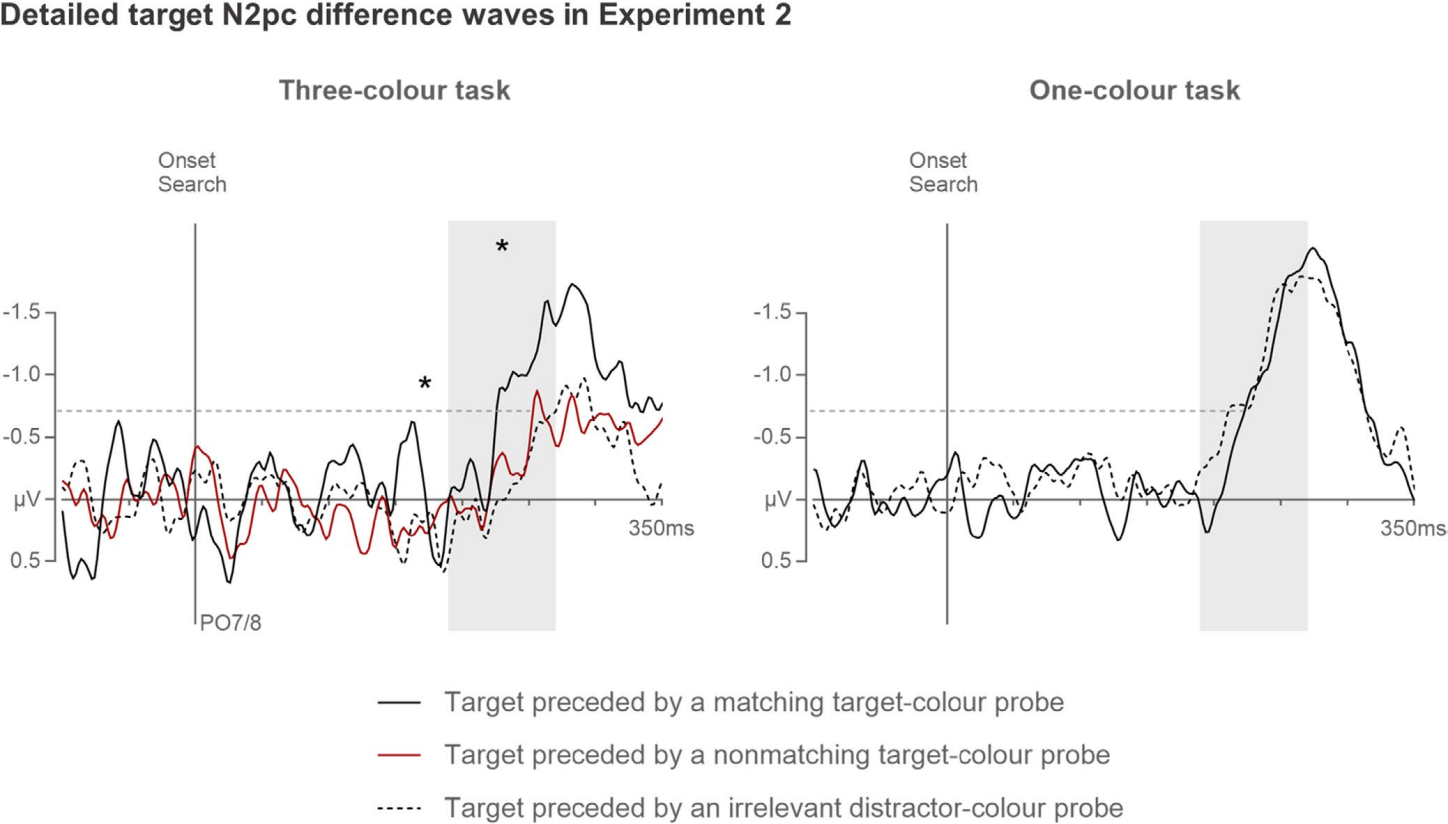
N2pc difference waveforms computed separately for targets that were preceded by color‐matching or non‐matching target‐color probes, or by irrelevant distractor‐color probes in the three‐color (left panel) and one‐color tasks (right panel) of Experiment 2. Shaded areas indicate N2pc time windows (190–270 ms after search display onset). Asterisks represent significant differences in mean amplitudes and onset latencies (measured at −0.7 μV, as indicated by the dashed horizontal lines).

Although this explanation may seem speculative, it can be directly tested based on the data obtained in Experiment 2. If the activation level of a particular color template in the three‐color task is temporarily enhanced by its match with a color singleton probe, this should have direct consequences for the attentional processing of search displays. More specifically, if a selection of probe 7 that immediately precedes a search display selectively boosts a specific template, this should benefit attentional guidance and target selection on trials where the subsequent search target matches this template. Such a benefit was indeed observed behaviourally for these trials by Kerzel and Grubert (2022) during three‐color search. To obtain more direct electrophysiological evidence for such probe‐target color match benefits, we conducted additional analyses of target N2pc components measured in the one‐color and three‐color tasks of Experiment 2. These N2pcs were computed separately for targets that either matched or did not match the color of the immediately preceding probe 7. Figure 7 (left panel) shows N2pc difference waveforms for targets in the three‐color task that were preceded either by a color‐matching or non‐matching target‐color probe or by an irrelevant distractor‐color probe. When the target was preceded by a matching target‐color probe, N2pcs were significantly larger (−0.9 μV), *t*(17) = 2.9, *p* = .040, *d* = 0.59, and emerged earlier (231 ms), *t*_c_(17) = 6.1, *p* < .001, *η*_pc_^2^ = .71, than when it was preceded by a non‐matching target‐color probe (−0.5 μV; 276 ms). In contrast, there were no N2pc amplitude or onset latency differences between trials in which targets were either preceded by a non‐matching target‐color or a distractor‐color probe (−0.4 μV, 270 ms), both *t*(17) < 1. This demonstrates that a color match between probe 7 and the subsequent target does indeed facilitate template‐guided target selection. Figure 7 (right panel) shows the corresponding target N2pc results for the one‐color task, separately for trials where the target was preceded by a target‐color probe (−1.0 μV, 225 ms) or by a distractor‐color probe (−1.0 μV, 215 ms). In this task, there were no N2pc amplitude or onset latency differences associated with the probe‐target color relationship, both *t*(17) < 1.1, demonstrating that target selection remained efficient regardless of whether probe 7 matched the color of the target or a distractor. Moreover, a comparison of trials where targets were preceded by a matching probe between the one‐color and three‐color tasks revealed no N2pc amplitude and latency differences, both *t*(17) < 1.

These N2pc results were mirrored by the behavioral data. RTs in the three‐color task were significantly faster when the target was preceded by a matching (724 ms) versus non‐matching target‐color probe (744 ms), *t*(17) = 3.0, *p* = .032, *d* = 0.20. There was no RT difference between trials in which the target was preceded by a non‐matching target‐color or a distractor‐color probe (732 ms), *t*(17) < 1. In the one‐color task, RTs were virtually identical on trials in which the target was preceded by a target‐color (615 ms) or a distractor‐color probe (616 ms), *t*(17) < 1. However, even though search was faster in the three‐color task when targets were preceded by color‐matching probes, RTs on these trials were still substantially slower than in the one‐color task, *t*(17) = 6.4, *p <* .001, *d* = 1.3.^4^ In other words, while the search costs associated with multiple‐ as compared to single‐color search on attentional guidance (as measured with the N2pc) can be fully accounted for by probe‐target color relationships, there are additional costs at the behavioral level which are likely to be generated at post‐guidance stages of attentional selectivity (see Ort et al., 2019).

Overall, these additional analyses support the hypothesis that between‐template competition during the three‐color search affects the efficiency of attentional guidance toward search targets and produces search performance costs relative to a single‐color search. In this context, it is interesting to note that there were no target N2pc differences between trials where the preceding probe matched a different target color (resulting in increased activation of a target non‐matching template) and trials where this probe matched a distractor color (producing no additional activation of any target‐color template). This suggests that while the prior activation of a matching template facilitates target selection in multiple‐color search, the activation of a different non‐matching template produced no additional costs.

In summary, the current study has obtained new insights into the nature of capacity limitations that arise when observers search for one of several possible target objects and multiple preparatory target templates have to be activated concurrently. First, and most importantly, the presence of reliable N2pc components to target‐color probes during a three‐color search when target identity was unpredictable, and the absence of any N2pcs to distractor‐color singleton cues, shows that at least three search templates can be activated in parallel. However, the template‐guided allocation of attention toward search targets, as well as search performance, are less efficient during three‐color as compared to one‐color search, and these costs are associated with inhibitory interactions between simultaneously active search templates.

## AUTHOR CONTRIBUTIONS

**Anna Grubert:** Conceptualization; formal analysis; funding acquisition; methodology; project administration; supervision; writing – original draft; writing – review and editing. **Ziyi Wang:** Data curation; formal analysis; visualization; writing – review and editing. **Ella Williams:** Data curation; writing – review and editing. **Mikel Jimenez:** Data curation; formal analysis; writing – review and editing. **Roger Remington:** Conceptualization. **Martin Eimer:** Conceptualization; funding acquisition; methodology; writing – original draft; writing – review and editing.

## FUNDING INFORMATION

This work was supported by research grants of the Leverhulme Trust (RPG‐2020‐319) awarded to AG and the Economic and Social Research Council (ES/V002708/1) awarded to ME.

## CONFLICT OF INTEREST STATEMENT

The authors report no conflict of interest.

## Supporting information


**Figure S1.** Grand‐averaged ERPs elicited at electrode sites PO7/8 contralateral and ipsilateral to 1‐back (top panel) and 2‐back (bottom panel) irrelevant target‐color probes 1–7 in Experiment 1. N2pc time windows are indicated by shaded areas (190–270 ms after the onset of each individual probe).
**Figure S2.** Grand‐averaged ERPs elicited at electrode sites PO7/8 contralateral and ipsilateral to target‐color probes in the one‐color (top panel) and three‐color tasks (bottom panel) of Experiment 2. N2pc time windows are indicated by shaded areas (190–270 ms after the onset of each individual probe).
**Figure S3.** Grand‐averaged ERPs elicited at electrode sites PO7/8 contralateral and ipsilateral to distractor‐color probes in the one‐color (top panel) and three‐color tasks (bottom panel) of Experiment 2. N2pc time windows are indicated by shaded areas (190–270 ms after the onset of each individual probe).

## Data Availability

The data supporting the findings of this study are available upon request.

## Footnotes

^1^
Note that without the combined power of the two task conditions, N2pc components to probe 4 in both the one‐color and three‐color task failed to reach significance, both *t*(17) < 1.3, *p* > .209, *d* = .06. All other *t* tests for probes 5–7 were significant, all *t*(17) > 2.7, *p* < .015, *d* > .23.

^2^
These amplitude results were the same when the target N2pcs were re‐analyzed in a time window of 240–320 ms that matched the time course of target N2pcs more closely (starting at 50% of the peak amplitude of the pooled one‐ and three‐color target N2pc). Target N2pcs were reliable, *F*(1,17) = 70.2, *p* < .001, *η*_p_^2^ = .81, and were significantly increased in the one‐color (−1.6 μV) as compared to the three‐color task (−0.8 μV), *F*(1,17) = 22.3, *p* < .001, *η*_p_^2^ = .57. For completeness, the target N2pc in Experiment 1 (−1.2 μV) was also reliable in this time window, *t*(17) = 7.5, *p* < .001, *d* > .32.

^3^
Note that power was sufficient to detect large behavioral and N2pc effects. Because the probe N2pcs contained fewer epochs than the RT averages and target N2pcs it is theoretically possible that small effects of task at the level of the probe N2pcs may have been missed. However, there was no statistical evidence for the existence of such effects (i.e., *F*‐values were <1 for all interactions between task and laterality).

^4^
Error rates were also still significantly increased in the three‐color (10%) as compared to the one‐color task (4%), *t*(17) = 3.7, *p* = .008, *d* = 1.2, even when the targets were preceded by target color‐matching probes. None of the other comparisons revealed significant differences in error rates, all *t*(17) < 1.
