# Supplementary material for: The capacity limitations of multiple‐template visual search during task preparation and target selection
Source: Psychophysiology. 2024 Nov 3;62(1):e14720. doi: 10.1111/psyp.14720 (PMC11775862; doi:10.1111/psyp.14720)
Supplement: Supplementary file 1 — Figure S1. Grand‐averaged ERPs elicited at electrode sites PO7/8 contralateral and ipsilateral to 1‐back (top panel) and 2‐back (bottom panel) irrelevant target‐color probes 1–7 in Experiment 1. N2pc time windows are indicated by shaded areas (190–270 ms after the onset of each individual probe). Figure S2. Grand‐averaged ERPs elicited at electrode sites PO7/8 contralateral and ipsilateral to target‐color probes in the one‐color (top panel) and three‐color tasks (bottom panel) of Experiment 2. N2pc time windows are indicated by shaded areas (190–270 ms after the onset of each individual probe). Figure S3. Grand‐averaged ERPs elicited at electrode sites PO7/8 contralateral and ipsilateral to distractor‐color probes in the one‐color (top panel) and three‐color tasks (bottom panel) of Experiment 2. N2pc time windows are indicated by shaded areas (190–270 ms after the onset of each individual probe). [file PSYP-62-e14720-s001.docx]

**Supplementary figures**


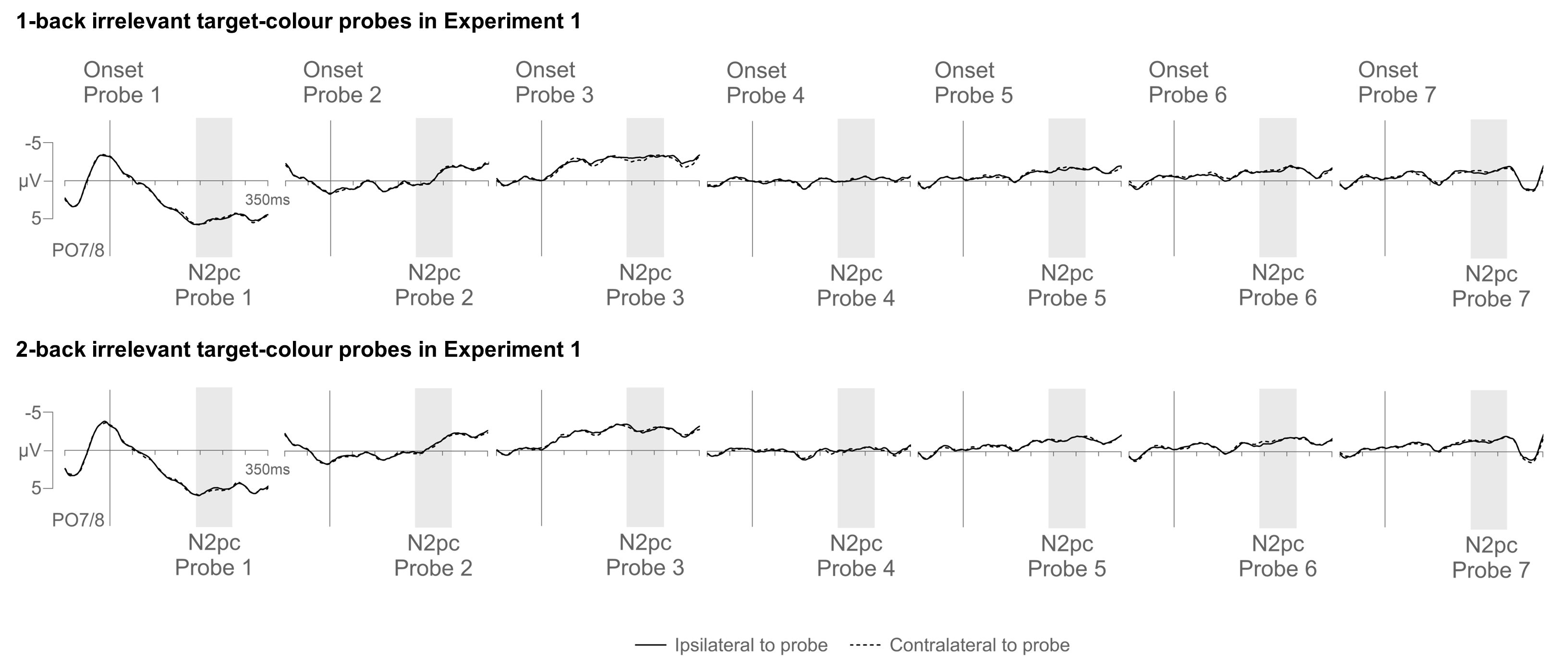


**Figure S1.** Grand-averaged ERPs elicited at electrode sites PO7/8 contralateral and ipsilateral to 1-back (top panel) and 2-back (bottom panel) irrelevant target-colour probes 1 to 7 in Experiment 1. N2pc time windows are indicated by shaded areas (190-270ms after onset of each individual probe).


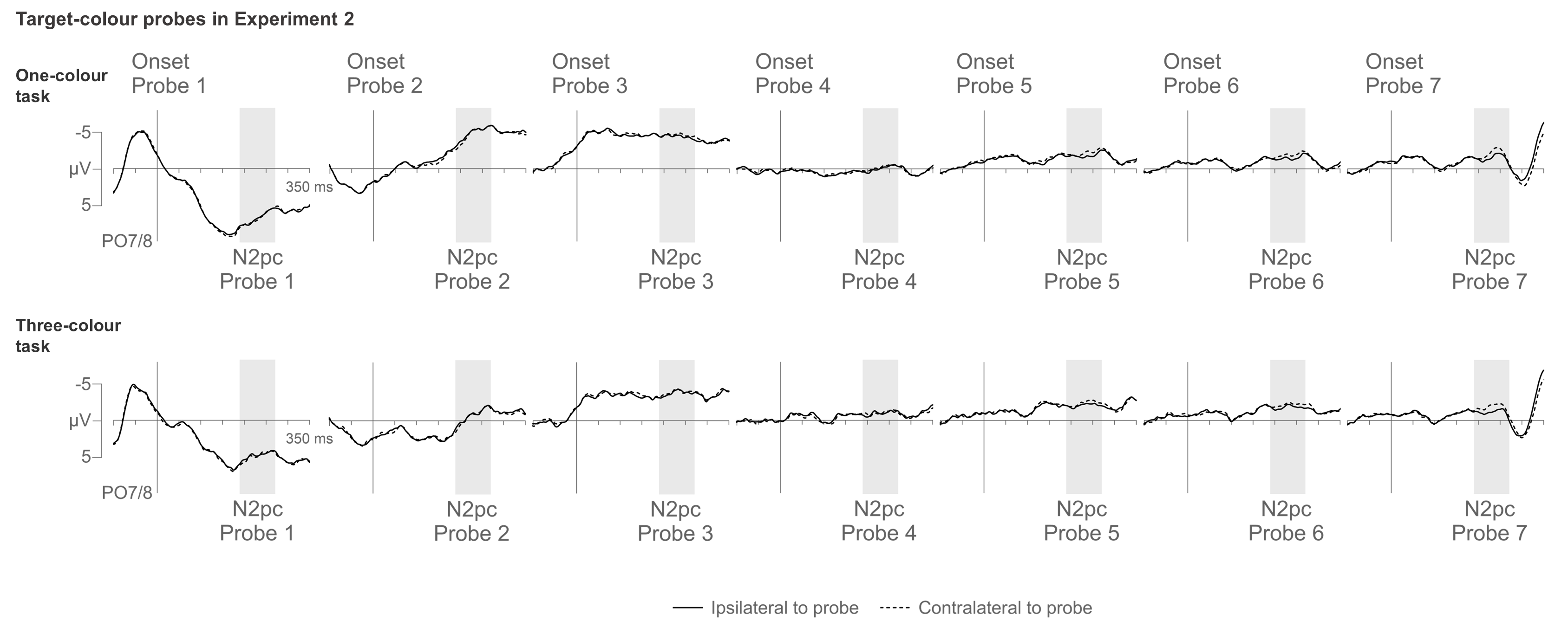


**Figure S2.** Grand-averaged ERPs elicited at electrode sites PO7/8 contralateral and ipsilateral to target-colour probes in the one-colour (top panel) and three-colour tasks (bottom panel) of Experiment 2. N2pc time windows are indicated by shaded areas (190-270ms after onset of each individual probe).


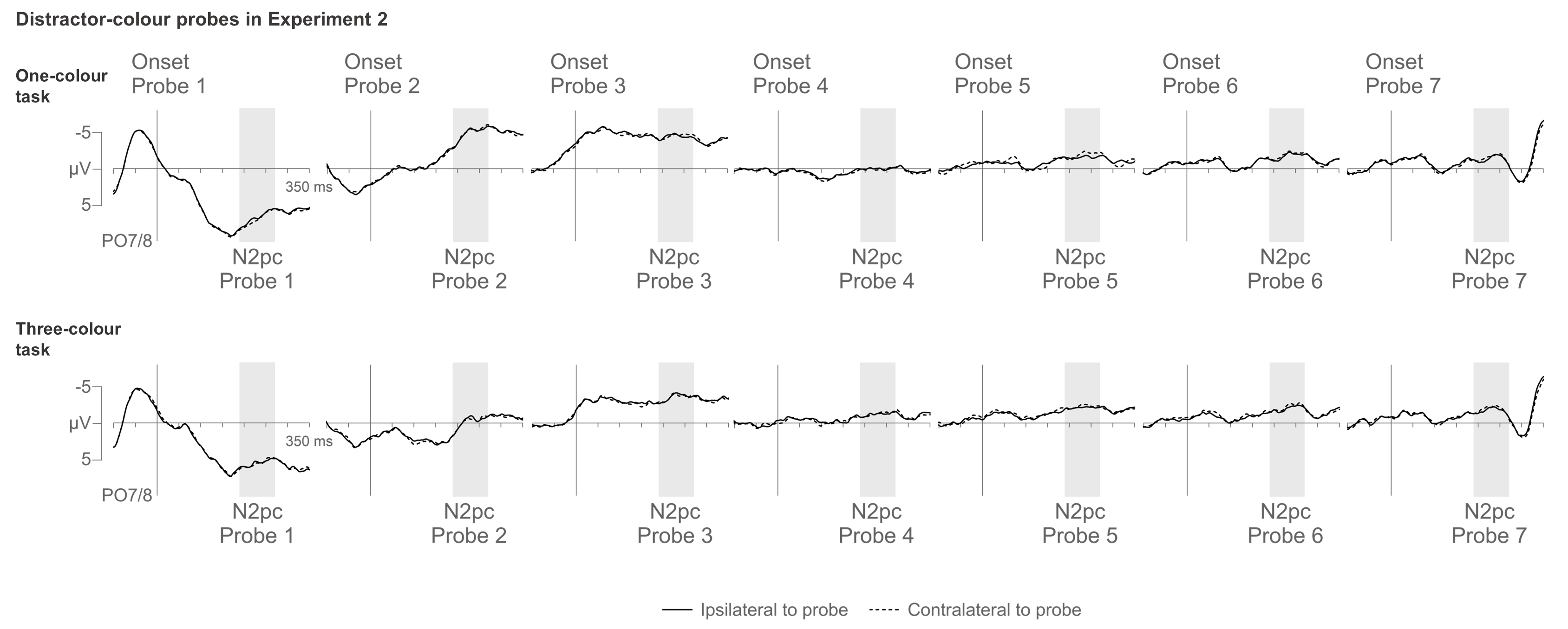


**Figure S3**. Grand-averaged ERPs elicited at electrode sites PO7/8 contralateral and ipsilateral to distractor-colour probes in the one-colour (top panel) and three-colour tasks (bottom panel) of Experiment 2. N2pc time windows are indicated by shaded areas (190-270ms after onset of each individual probe).
